# Ⅲa期非小细胞肺癌术后辅助治疗的临床应用

**DOI:** 10.3779/j.issn.1009-3419.2010.04.17

**Published:** 2010-04-20

**Authors:** 霞 张, 斌 张, 亚杰 高

**Affiliations:** 1 116021 大连，大连医科大学第一附属医院肿瘤科 Department of Medical Oncology, Dalian Medical University First Hospital, Dalian 116021, China; 2 116027 大连，大连医科大学第二附属医院肿瘤科 Department of Medical Oncology, Dalian Medical University Second Hospital, Dalian 116027, China

**Keywords:** 肺肿瘤, 放疗, 化疗, 综合治疗, Lung neoplasms, Radiotherapy, Drug therapy, Combined modality therapy

## Abstract

**背景与目的:**

Ⅲa期非小细胞肺癌（non-small cell lung caner, NSCLC）单纯根治性手术获益有限，目前倡导多学科综合治疗，但术后放疗的价值仍不清楚。本文比较分析Ⅲa期NSCLC术后化疗和术后放疗+化疗的生存期及副反应。

**方法:**

分析2003年12月-2007年6月大连医科大学第一附属医院收治的具有完全随访资料的Ⅲa期NSCLC患者52例。术后化疗组23例，术后放疗+化疗组29例。术后化疗组采用铂类为主的联合化疗方案，联合化疗药包括吉西他滨、长春瑞滨、多西他赛，化疗共4周期。术后放疗+化疗组采用序贯放化治疗，化疗2-4周期后加用放射治疗，放射治疗采用三维适形放疗（3D-CRT），靶区剂量50 Gy，化疗共4周期，化疗方案同单纯化疗组。观察Ⅲa期非小细胞肺癌术后辅助治疗对生存期的影响及毒副作用，分析疾病进展原因。

**结果:**

术后化疗组、术后放化疗组中位生存时间为32.5个月*vs* 31.9个月，差异无统计学意义（*P*=0.371）。两组中位无进展生存时间为11.0个月*vs* 17.0个月，差异有统计学意义（*P*=0.044）。两组1、2、3年生存率为87%、61%、33% *vs* 93%、69%、45%。不良反应主要表现在消化道和血液学方面，即恶心、呕吐及白细胞下降，两组间差异无统计学意义（*P* > 0.05）；放射性食管炎发生率为17.2%，急性及晚期放射性肺损伤发生率分别为13.8%和27.6%，均为Ⅱ级以下。疾病进展原因主要为远处转移，局部复发率及转移发生率两组比较差异无统计学意义（*P* > 0.05）。

**结论:**

Ⅲa期NSCLC治疗要以综合治疗为主；放疗的介入延长了无进展生存时间，并未显著增加治疗的毒副作用。

## Abstract (en)

**Background and objective:**

The efficacy of complete resection of the cancer for patients with stage Ⅲa non-small cell lung cancer (NSCLC) is limited. Synthetic therapy is taken the lead in advocating at present. However, the value of post-operative radiotherapy is not still clear. The aim of this study is to evaluate the survival time and side effects of postoperative chemotherapy or chemoradiotherapy in the treatment of stage Ⅲa NSCLC.

**Methods:**

Between December 2003 and June 2007, 52 cases that have completed followed-up data with stage Ⅲa of NSCLC received in the First Affiliated Hospital of Dalian Medical University. Twenty-three patients received postoperative chemoradiotherapy (group A) and 29 patients received postoperative chemotherapy combined with radiotherapy (group B). Group A adopted platinum-based combination chemotherapy for 4-6 cycles. The chemotherapeutics included gemcitabine, vinorelbine and docetaxel. Group B used chemotherapy for 2-4 cycles and then received 3-dimensional conformal radiotherapy (3D-CRT). The prescribe dose of target volume was 50 Gy. The chemotherapy was same as for group A and needed 4 cycles in all. The impact of postoperative adjuvant treatment on survival and toxicity was observed in patients with stage Ⅲa NSCLC and the reason of disease progression was analyzed.

**Results:**

The median survival was 32.5 months in group A and 31.9 months in group B (*P*=0.371). Progression-free survival extended about 6 months (*P*=0.044). The survival rate was 87% at 1 year, 0.1% at 2 year, 33% at 3 year for group A compared with 93%, 69%, 45% for group B. The major side effects were hematological and gastrointestinal toxicities, including nausea, vomiting and neutropenia. There was no significant difference in these toxicities between the two groups (*P* > 0.05). Radioactive esophageal infection occurred in 17.2% of the patients. Acute and late radioactive lung infection occurred in 13.8% and 27.6% of the patients. All these toxicities were below degree 2. Distant metastases were the main reason of disease progression. There was no significant difference in the rates of local recurrence and metastases between the two groups (*P* > 0.05).

**Conclusion:**

Combined modality therapy should be the main therapy of stage Ⅲa NSCLC. The addition of radiotherapy can effectively prolong progression-free survival and don't highly increase the toxicities.

外科切除是可切除Ⅲa期非小细胞肺癌（non smallcell lung cancer, NSCLC）的“标准治疗”，单纯完全切除术后5年生存率约为23%，目前的证据支持术后辅助化疗。术后放疗可以增加局部控制率，但局部复发率的降低并没有转换为生存期的延长，*meta*分析显示术后放疗不增加Ⅲ期NSCLC的死亡风险，但也不减少死亡危险，故目前临床推荐术后放疗方面有一定争议^[1]^。临床应权衡治疗的获益程度及可能毒性反应进行治疗。现将我院治疗经验总结如下，为临床工作提供参考。

## 1 材料与方法

### 1.1 病例选择

分析2003年12月-2007年6月大连医科大学第一附属医院收治的具有完全随访资料的Ⅲa期NSCLC病例共52例，其中手术+化疗组23例，手术+放化疗组29例。所有患者均经病理学诊断证实为NSCLC; 治疗前均行肺部CT、上腹部CT或B超、头部MRI、骨ECT等排除远处转移。并行血常规、血液生化检查及心电图等检查保证无放化疗禁忌。临床分期采用UICC 1997年颁布的分期标准。Karnofsky评分 > 70，预计生存 > 12周。病例分组情况详见表 1。

**1:** 患者临床资料 Clinical characteristics of patients

Characteristic	Operation+Chemotherapy group	Operation+Chemoradiation group	χ^2^	*P*
Gender			3.008	0.083
Male	17	19		
Female	6	10		
Age (years)			1.492	0.222
> 60	15	14		
< 60	8	15		
Histology			2.329	0.503
Squamous cell carcinoma	10	11		
Adenocarcinoma	7	13		
Adenosquamous Carcinoma	3	4		
Others	3	1		
Location			0.119	0.730
Center	9	10		
Surounding	14	19		
T Stage			4.636	0.098
T1	1	5		
T2	15	21		
T3	7	3		
N Stage			2.623	0.105
N1	2	0		
N2	21	29		
Style of surgery			1.279	0.258
Total pneumonectomy	5	3		
Pulmonary lobectomy	18	26		
Chemotherapy protocol			0.204	0.903
GP	3	3		
NP	9	13		
PD	11	13		

### 1.2 辅助治疗方法

#### 1.2.1 化疗

化疗采用以顺铂（DDP）为主的联合化疗方案，DDP 60 mg/m^2^-75 mg/m^2^，分2 d-3 d应用。联合新药吉西他滨（GEM）、长春瑞滨（NVB）、多西他赛（DOC）。分别为GP[GEM 800 mg/(m^2^, d)-1 000 mg/(m^2^, d)，d1、d8应用]、NP[NVB 20 mg/(m^2^, d)-25 mg/(m^2^, d)，d1、d8应用]、DP[DOC (60-75) mg/m^2^，d1应用]方案，所有患者均化疗共4周期。术后放化疗组在手术后行2-4周期化疗，继行放疗一疗程，放疗结束部分病例再行化疗，完成4周期。化疗方案GP 6例（11.5%）、NP 22例（42.3%）、DP 24例（46.2%）。

#### 1.2.2 放疗

模拟定位机下体模固定，确定扫描中心后CT扫描，范围自下颌骨下缘至膈肌以下，包括肾上腺。CT图像传输至GE-SIM影像工作站勾画靶区，参考术前CT、PET/CT、手术记录等划定治疗靶区。靶区包括纵隔淋巴结受累区，术后病理虽阴性但术前高度可疑或外科认为淋巴结清扫未彻底者的淋巴引流区域。对右中下叶或左舌叶、左下叶病变、纵隔淋巴结受侵犯者，隆突下淋巴结包入CTV; 对于左上叶病变，如果纵隔淋巴结及隆突下淋巴结受侵，则主动脉窗淋巴结包入CTV; 如果隆突下淋巴结或者纵隔淋巴结受侵犯者，同侧肺门包入CTV。PTV为CTV外放1.0 cm。

采用共面和（或）非共面照射野，以PTV几何中心为等中心点，应用2-4个野适形照射，剂量计算后进行均一性校正，使95%的等剂量线三维包绕靶区，经剂量体积直方图（DVH）评价。肺组织受量用V20作评估，一般V20限制≤25%，脊髓要求最大剂量点低于45 Gy。食管最大剂量点低于60 Gy。心脏V40≤40%-50%。如多次缩野最后评估需将各分次计划叠加融合计算各正常组织总受量。

计划实施使用VARIAN 23EX直线加速器，6 MV-X线照射，常规分割，照射剂量1.8 Gy/次-2.0 Gy/次，5次/周，总剂量50 Gy。

### 1.3 评价方法

总生存时间（overall survival, OS）定义为患者开始治疗至患者死亡或末次随诊时间。无进展生存时间（progression-free survival, PFS）定义为患者开始治疗至疾病进展或疾病尚未进展的末次随访时间。

所有病例评价不良反应，不良反应评价按WHO标准分为0-IV级，正常组织放射治疗副反应按美国肿瘤放射治疗协作组RTOG诊断分级标准分别评价急性放射性肺炎、晚期放射性肺炎及放射性食管炎分0-IV级。

### 1.4 统计学处理

统计学分析采用SPSS 16.0软件，用率和百分比来描述记数资料，组别比较时记数数据用χ^2^检验，等级资料采用秩和检验，生存分析采用*Kaplan-Meier* 和*Log-rank*方法，以*P* < 0.05为有统计学差异。

## 2 结果

### 2.1 远期疗效

对所有患者进行随访，截止2009年7月，随访6.3个月-64.8个月，中位随访28.5个月。手术+化疗组、手术+放化疗组中位生存时间为32.5个月*vs* 36.5个月，无统计学差异（*P*=0.371）。但两组中位无进展生存时间为11.0个月*vs* 17.0个月，差异有统计学意义（*P*=0.044）。两组生存率及生存曲线见表 2、图 1、图 2。

**2:** 两组患者的远期疗效（%） The long-term efficacy of patients in two groups (%)

Group	Survival rate		
	1-year	2-year	3-year
Operation+Chemotherapy group	87%	61%	33%
Operation+Chemoradiation group	93%	69%	45%

**1:**
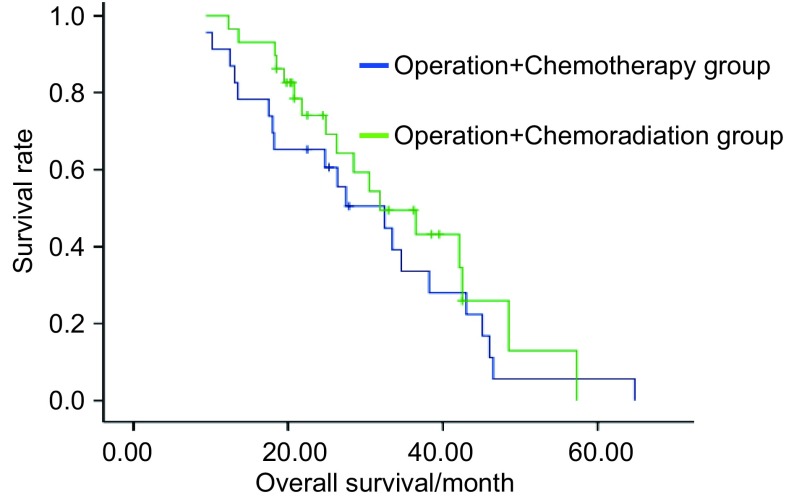
两组患者的远期疗效 The long-term efficacy of patients in two groups

**2:**
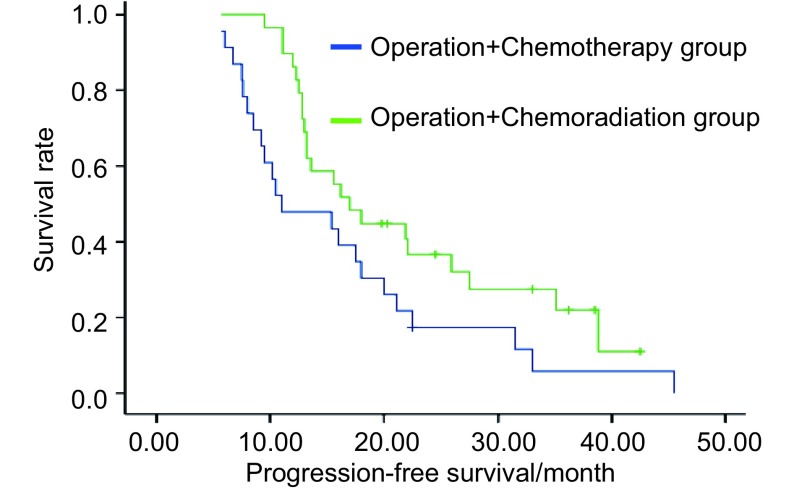
两组患者的无进展生存曲线 Progression-free survival curves of patients in two groups

### 2.2 不良反应

52例患者的不良反应主要表现在血液学毒性和消化道反应，血液学毒性主要表现为白细胞减少，手术+化疗组Ⅰ-Ⅲ度白细胞减少的发生率为73.9%，而手术+放化疗组为65.5%，经χ^2^检验，差异无统计学意义（*P* > 0.05）; 消化道反应主要恶心、呕吐，手术+化疗组Ⅰ-Ⅲ度反应的发生率为69.6%，手术+放化疗组为79.3%，经χ^2^检验，差异无统计学意义（*P* > 0.05）; 与放疗有关的放射性食管炎发生率为（5/29）17.2%，基本于放疗开始后2-6周发生，均为Ⅱ度以下。急性放射性肺炎发生率为（4/29）13.8%，多于放疗开始后3-6周急性发生，均为Ⅰ-Ⅱ级，放疗结束后6个月复查，随访放射性肺损伤，发现8例发生影像学改变，发生率为27.6%（表 3）。

**3:** 52例患者的不良反应 Toxicities of 52 patients

Adverse reaction	Operation+Chemotherapy group				Operation+Chemoradiation group			Incidence rate
	Ⅰ	Ⅱ	Ⅲ		Ⅰ	Ⅱ	Ⅲ	
Decrease of leucocyte	12	3	2		14	3	2	69.2%
Decrease of platelets	3	0	0		3	1	0	13.4%
Decrease of hemoglobin	6	1	0		7	2	0	30.8%
Nausea and vomiting	10	5	1		12	8	3	75.0%
Liver function damage	3	1	0		4	1	0	17.3%

### 2.3 进展分析

共有44例患者在不同时期出现疾病进展，死亡37例，71.2%的患者术后2年内出现疾病进展。其中单纯局部复发（或进展）者共11例，单纯远处转移共26例，局部复发（或进展者）伴远处转移共7例，观察手术+化疗组、手术+放化疗组局部复发及远处转移率差异无统计学意义（*P* > 0.05），死亡原因多为远处转移所致的各器官功能障碍（表 4）。

**4:** 52例患者复发转移特点 Reccurence and metastasis characteristics of 52 patients

Location	Operation+Chemotherapy group	Operation+Chemoradiation group	χ^2^	*P*
Local reccurence (or progression)	7	4		
Brain metastasis	1	2		
Bone metastasis	2	3		
Lung metastasis	3	4		
Adrenal gland metastasis	0	1		
Liver metastasis	2	2		
Non region lymphatic metastasis	1	0		
Pleura metastasis	0	0		
Multiple sites metastasis	4	3		
Local reccurence (or progression) and metastasis	2			
Local progression ratio	30.4%	13.8%		
Metastasis ratio	69.8%	86.2%	1.091	0.296

## 3 讨论

肺癌为高发肿瘤，其中75%为NSCLC。外科手术、放射治疗和化学治疗仍为NSCLC的三大治疗手段，但由于大多数患者在诊断时已是局部晚期或有远处转移，故整体的5年生存率仍较低，其原因主要是肿瘤在早期即有全身播散倾向，诊断时超过50%的病例不适于接受外科治疗。即使是完全切除术后，仍有相当部分的患者最终死于肿瘤复发转移^[1]^。Ⅲ期NSCLC约占40%，其中潜在手术机会的Ⅲa期较少，多数为不可手术的Ⅲb期。应如何治疗仍有争议，特别是对Ⅲa（N2）期NSCLC。临床N2的局部晚期NSCLC，其自然中位生存期为7个月，即使手术后5年生存率也不到10%。大宗病例的回顾性研究^[2]^提示，对于病理N2的患者，单纯手术治疗的疗效也不理想，5年生存率仅为20%-25%。因此，单纯根治性手术获益有限，国内外学者均倡导多学科综合治疗。Ⅲa病例虽然单纯手术后复发率和死亡率高，但术后放疗的价值仍不清楚。目前认为N2或T3-4N1病例根治术后需要进行计划性临床研究（包括放射治疗和化疗）。采用三维适形放射治疗技术，明确治疗体积，优化剂量分布以降低肺和心脏的受照射体积和照射剂量^[3]^。

回顾国内文献，汪虹等^[4]^报告107例Ⅲa期NSCLC的临床研究结果表明，术后放疗加化疗组5年生存率为34.6%，单纯手术组为20.8%，两组比较无统计学差异（*P* > 0.05）。但是多因素分析显示，N分期是生存率的独立预后因素，并且是远处转移的高危因素，认为N2分期患者应采用术后放疗联合化疗。潘泓等^[5]^报道Ⅲ期NSCLC切除术后予顺铂为主的化疗，可提高长期生存，5年生存率为27.6%。放疗作为术后辅助治疗的方法也被充分认可，并广泛应用于临床。李昉等^[6]^研究表明，手术后放化疗组患者的1、3、5年生存率分别为86.6%、37.8%、32.3%，与单纯手术组的84.6%、13.6%、9.1%比较有统计学差异。林原等^[7]^报道51例Ⅲa期NSCLC患者术后3D-CRT联合化疗1、2年生存率分别为93%、70%。认为术后放疗加化疗有可能提高Ⅲ期NSCLC的生存率。

本组研究中，术后化疗组、术后放化疗组中位生存时间为32.5个月*vs* 31.9个月，差异无统计学意义（*P*=0.371）。两组中位无进展生存时间为11.0个月*vs* 17.0个月，差异有统计学意义（*P*=0.044）。提示术后辅以放疗，可延长无进展生存时间，更大程度上控制了疾病进展。但对总生存时间的延长无明确改善，仍有待长期随访并增大样本量证实，术后化疗组1、2、3年生存率为87%、61%、33%，术后放化疗组为93%、69%、45%。生存率与国内同期文献报道相仿。

肺癌放疗的毒副作用主要表现为放射性肺炎及放射性食管炎。尽管理论上讲，3D-CRT治疗NSCLC较常规放疗明显降低了正常肺组织的高剂量照射容积，但其所致的放射性肺损伤仍不可忽视。与常规放疗所不同的是我们可以依据MLD、V20、V30、NTCP等肺受照剂量参数进行放射性肺损伤可能性预测，而且大量研究V20、NTCP等被作为3D-CRT治疗NSCLC发生放射性肺炎的预测参数，但它们各自与放射性肺炎的关系不同的研究有不同的结果。Graham等^[8]^通过99例不能手术的NSCLC患者3D-CRT后放射性肺炎的发生与V20、NTCP、有效容积，肺平均受照剂量的关系，多因素分析结果显示，仅V20是≥2级放射性肺炎密切相关因素。≥3级的肺炎均发生在V20≥35%的病例，控制V20≤20%可明显减少急性放射性肺炎的发生。本组研究中放疗剂量为50 Gy，肺V20控制于17.2%-25.2%，中位23.6%。急性及晚期放射性肺损伤发生率分别为13.8%和27.6%，均为Ⅱ级以下。提示V20≤25%基本保证治疗安全。

影响放射性食管炎的发生因素因不同研究方向和内容，结果不尽相同，但食管最大受量的研究基本肯定。如2003年Singh等^[9]^报告了207例不能手术肺癌患者的放疗结果，所有患者的总剂量≥60 Gy，当食管最大剂量超过58 Gy时，≥Ⅲ级的食管损伤会明显提高（多因素分析，*P*=0.001）。本组食管最大受照剂量为3 587 cGy-5 235cGy，中位4 635 cGy。放射性食管炎发生率17.2%。反应程度亦在临床可承受范围内。所有发生放射性食管炎病例其食管最大受照剂量均超过50 Gy，提示最高剂量的限制可影响放射性食管炎的发生。

对于疾病复发进展的研究，各家报道不一，罗扬等^[10]^总结231例Ⅱ期和Ⅲ期NSCLC根治术后生存情况和复发转移特点，发现124例（54.5%）出现复发转移，其中82.5%的首发部位为远处转移，92.8%的复发转移出现在术后2年内。李昉等^[6]^研究中提出术后5年转移率放化疗组为50%。本研究中71.2%的患者术后2年内出现疾病进展者。其中单纯局部复发者共11例，单纯远处转移共26例，局部复发伴远处转移共7例，观察手术+化疗组、手术+放化疗组局部复发及远处转移率差异无统计学意义（*P* > 0.05），一定程度上提示我们对于肺癌这种高度恶性肿瘤，联合放疗并未降低局部复发率，死亡原因仍为远处转移，全组转移率为75.0%。对于术后联合放化疗病例无进展生存时间延长，而局部复发率并未见减低的原因分析，可能与原发灶的整体控制而推迟了复发及转移的时间有关。

Ⅲa期NSCLC术后辅助治疗的临床工作任重道远，争议较多，临床工作中应结合每一例患者的年龄、肺功能基储体力状况评分及合并症，选择适合该患者的治疗方案，不要一味地追求强烈的多种治疗方法联合应用，避免过度治疗。
